# In vitro antibacterial activity of thymol and carvacrol and their effects on broiler chickens challenged with *Clostridium perfringens*

**DOI:** 10.1186/s40104-015-0055-7

**Published:** 2015-12-24

**Authors:** Encun Du, Liping Gan, Zhui Li, Weiwei Wang, Dan Liu, Yuming Guo

**Affiliations:** State Key Laboratory of Animal Nutrition, College of Animal Science and Technology, China Agricultural University, Beijing, 100193 P. R. China

**Keywords:** Thymol, Carvacrol, Essential oils, *Clostridium perfringens*, *Escherichia coli*, Broiler chickens

## Abstract

**Background:**

In the post-antibiotic era, essential oils (EO) are promising alternatives to growth-promoting antibiotics. The aim of the present study was to investigate the antibacterial activities of an EO product and its components thymol and carvacrol in vitro, and the efficacy of EO to control *Clostridium perfringens* challenge in broiler chickens.

**Results:**

The in vitro minimum inhibitory concentration assay showed strong antibacterial activity of the EO product, thymol, and carvacrol against pathogenic *Escherichia coli*, *C. perfringens*, and *Salmonella* strains, and weak activity towards beneficial *Lactobacillus* strains. Besides, an additive effect was observed between thymol and carvacrol. The in vivo study was carried out with 448 male broiler chicks following a 4 × 2 factorial arrangement to test the effects of EO supplementation (0, 60, 120, or 240 mg/kg EO in wheat-based diet), pathogen challenge (with or without oral gavage of *C. perfringens* from day 14 to day 20) and their interactions. Each treatment consisted of eight replicate pens (seven birds/pen). The challenge led to macroscopic gut lesions, and resulted in a significant increase in ileal populations of *C. perfringens* and *Escherichia* subgroup (*P ≤* 0.05) on day 21. Dietary EO supplementation did not influence *C. perfringens* numbers, but linearly alleviated intestinal lesions on day 21 and 28 (*P =* 0.010 and 0.036, respectively), and decreased *Escherichia* populations in ileum with increased EO dosages (*P* = 0.027 and 0.071 for day 21 and 28, respectively). For caecum, EO quadratically influenced *Lactobacillus* populations on day 21 (*P* = 0.002), and linearly decreased the numbers of total bacteria and *Escherichia* on day 28 (*P* = 0.026 and 0.060, respectively). Mean thymol and carvacrol concentrations in the small intestine were 0.21 and 0.20 μg/g in intestinal digesta (wet weight), respectively, for birds fed 60 mg/kg EO, and 0.80 and 0.71 μg/g, respectively, for birds fed 240 mg/kg EO.

**Conclusions:**

These results indicated that dietary EO supplementation could affect intestinal microbiota and alleviate intestinal lesions in broilers, which may contribute in controlling *C. perfringens* infection in broiler chickens.

## Background

*Clostridium perfringens* is a Gram-positive, anaerobic, spore-forming bacterium, which is usually classified into five toxinotypes (A, B, C, D, and E) according to the production of four major toxins, called alpha, beta, epsilon and iota. Although *C. perfringens* is a commensal bacterium of the intestine, *C. perfringens* type A is considered the main causative agent of necrotic enteritis (NE) in poultry [1, 2]. Proliferation of pathogenic *C. perfringens* and released toxins, especially NetB toxin, result in NE in poultry [2, 3]. NE is a widespread disease in poultry, as estimated to cost the international poultry industry approximately two billion US dollars annually [4]. Impaired feed digestion and absorption, reduced growth rate, and mortality are the major reasons for production losses associated with NE [2, 5].

The use of in-feed antibiotics has been the main strategy for controlling NE in poultry. However, public concern about the threat of antibiotic-resistant pathogens has forced the poultry industry to consider alternatives to antibiotics for poultry production [5]. Probiotics, prebiotics, organic acids, enzymes, and essential oils (EO) are among the alternatives [5]. Also, because of consumer preference for natural products, the use of EO has increased appeal [6].

The antibacterial properties of EO have long been recognized and widely tested in vitro against a wide range of pathogenic bacteria, including both Gram positive and Gram negative bacteria [6–8]. Although the antibacterial mechanism of EO and their constituents is not fully understood, studies have shown that constituents with a phenolic structure, such as eugenol, carvacrol and thymol have the greatest bactericidal activities, followed by aldehydes, ketones, alcohols, ethers and hydrocarbons [9–11]. However, it seems that the efficacy of EO is not consistent in vivo: both improved and unchanged growth performance and intestinal microbiota have been reported in pigs and chickens [6, 8, 12–14]. Actually, it is difficult to compare the efficacy of EO considering the fact that EO blends containing various constituents have been used in vivo in published reports [6, 8, 12–15]. In addition, little information is available regarding the relationship between antibacterial activities in vitro and in vivo. Therefore, the aim of the present study was to investigate the efficacy of an EO product as well as its components thymol and carvacrol on pathogenic bacteria and benefical *Lactobacillus* strains, and to investigate the effects of EO on broiler chickens challenged with *C. perfringens*. In addition, thymol and carvacrol concentrations in intestinal digesta were assayed.

## Methods

### In vitro study

#### Chemicals

Thymol and carvacrol were purchased from Sigma-Aldrich Corporation (St Louis, MO, USA), with compound purities at ~ 98 %. A commercial EO product was provided by Novus International Inc. (St Charles, MO, USA), which contained 25 % thymol and 25 % carvacrol as active components, 37 % silicon dioxide as a caking inhibitor, and 13 % glycerides as stabilizing agents.

### Preparation of cultures

Thymol, carvacrol and the commercial EO product were individually tested against a panel of undesirable bacteria and beneficial *Lactobacillus* strains. The undesirable bacteria included two chicken *Escherichia coli* field strains CVCC1553 and CVCC1490 (serotype O78), chicken *C. perfringens* field strains (CVCC2027 and CVCC2030), *Salmonella enterica* serovar Typhimurium (CVCC541), *Salmonella enterica* serovar Enteritidis (CVCC2184) and *Salmonella enterica* serovar Pullorum (C79-13), which were obtained from China Veterinary Culture Collection Center (Beijing, China). Another *S.* Enteritidis field strain (ATCC13076) was obtained from American Type Culture Collection (Manassas, VA, USA). In addition, three strains of *Lactobacillus* were tested. *L. acidophilus* (GIM1.730) was obtained from Guangdong Microbiology Culture Center (Guangdong, China), and *L. reuteri* and *L. salivarius* were isolated from the gastrointestinal content of healthy broiler chickens by our laboratory staff.

Overnight cultures of *E. coli* and *Salmonella* were prepared freshly by cultivation from frozen stock at 37 °C in Luria-Bertani broth with continuous shaking. Cultures of *C. perfingens* were prepared anaerobically at 37 °C overnight in cooked meat medium and Man-Rogosa-Sharpe (MRS) broth used for growing of *Lactobacillus* at 37 °C without shaking.

### Minimum inhibitory concentration and minimal bactericidal concentration assay

The minimum inhibitory concentration (MIC) was determined via a conventional broth dilution method, as described by Ivanovic et al. with some modifications [16]. Briefly, the commercial EO product, thymol, and carvacrol were initially dissolved as 300 mg/mL stock solution in dimethylsulfoxide (DMSO) and diluted to 6 mg/mL in Mueller-Hinton broth with vigorous shaking. Then, two-fold serial dilutions of thymol and carvacrol were prepared, producing concentrations at 6000, 3000, 1500, 750, 375, 187.5, 93.75 and 46.875 μg/mL. For the detection of *Lactobacillus,* MRS broth was used for dilution. In order to avoid the antibacterial effects of DMSO itself, the final DMSO concentrations never exceeded 2 % (by volume). Then, each diluted broth was inoculated with fresh microbial suspension (final concentration: 1 ~ 5 × 10^5^ colony-forming units (cfu)/mL). *E. coli* and *Salmonella* were incubated at 37 °C with continuous shaking overnight. *C. perfringens* and *Lactobacillus* were incubated anaerobically at 37 °C for 24 and 16 h, respectively, without shaking. A positive growth control containing 2 % DMSO without EO or components, and a negative control containing no bacteria were included in each experiment. After incubation, the optical density (OD) of suspension was measured using a spectrophotometer at 595 nm. In addition, 50 μL from each broth dilution was inoculated for enumeration in duplicate onto nutrient agar for *E. coli* and *Salmonella*, sulfite-polymyxin-sulfadiazine agar for *C. perfringens*, and MRS agar for *Lactobacillus,* all by the spread plate method. MIC was defined as the lowest concentration of EO or components that showed no increase in OD following incubation. The minimal bactericidal concentration (MBC) was defined as the lowest concentration of EO or components with which no viable bacteria were detected. All assays were performed in triplicate.

### Combination assay

A combination assay between thymol and carvacrol was performed by the checkerboard method, as previously described [17]. Two-fold serial dilutions of one component were tested in the presence of serial concentrations of the other component (which did not inhibit bacterial growth alone). The fractional inhibitory concentration (FIC) was calculated as follows: FIC of component A  = MIC of component A in combination divided by the MIC of component A alone, FIC of component B  = MIC of component B in combination divided by the MIC of component B alone, and FIC index (FICI) = FIC of component A + FIC of component B. An FICI < 0.5 was considered to demonstrate synergy. When an FICI fell between 0.5 and 1.0, it was defined as an additive effect and, between 1.0 and 4.0, it was classified as no interaction. Finally, an FICI > 4.0 indicated antagonism between the components in a combination.

### In vivo study

#### Birds, diets, and experimental design

All experimental procedures were approved by the China Agricultural University Animal Care and Use Committee. A total of 448 one-day-old male broiler chicks (Cobb 500) were used for a 28-day experiment. Chicks were assigned to eight treatments, following a 4 × 2 factorial arrangement in a randomized complete block design to evaluate dietary EO supplementation (0, 60, 120, or 240 mg/kg EO in wheat-based diet), pathogen challenge (with or without oral gavage of *C. perfringens* from day 14 to 21) and their interactions. Each treatment consisted of eight replicate pens (seven birds/pen). The EO used in this trial was the commercial product mentioned above, which contained 25 % thymol and 25 % carvacrol as active components. Chickens were fed starter (day 0–21) and finisher (day 21–28) diets in the form of mash and had access to feed and water *ad libitum*. Proliferation of *C. perfringens* was promoted by formulating antibiotic-free and coccidiostat-free wheat-based diets. All nutrients were formulated to meet or exceed the feeding standard of China (NY/T 2004) for broilers [18] (Table 1).

**Table 1:** Diet composition and nutrient levels

Item (%, unless otherwise indicated)	Starter diets (day 0–21)	Grower diets (day 22–28)
Ingredient		
Wheat	62.75	68.5
Soybean meal	29.61	23.72
Soybean oil	3.40	4.00
Dicalcium phosphate	1.91	1.63
Limestone	1.04	0.96
Sodium chloride	0.35	0.35
Choline chloride (50 %)	0.25	0.25
L-Lysine (99 %)	0.25	0.24
DL-Methionine (98 %)	0.19	0.11
Antioxidants	0.03	0.03
Trace mineral premix ^a^	0.20	0.20
Vitamin premix ^b^	0.02	0.02
Calculated nutrient levels		
Metabolizable energy (Mcal/kg)	2.90	2.98
Protein	21.00	19.00
Calcium	1.00	0.90
Available phosphorus	0.45	0.40
Lysine	1.15	1.00
Methionine	0.50	0.40

^a^ The trace mineral premix provided the following (per kilogram of diet): manganese, 100 mg; zinc, 75 mg; iron, 80 mg; copper, 8 mg; selenium, 0.25 mg; iodine, 0.35 mg

^b^ The vitamin premix provided the following (per kilogram of diet): vitamin A, 18750 IU; vitamin D_3_, 3750 IU; vitamin E, 28 IU; vitamin K_3_, 3.975 mg; thiamine mononitrate, 3 mg; riboflavin, 9 mg; vitamin B_12_, 0.0375 mg; d-biotin, 0.150 mg; folic acid, 1.875 mg; d-calcium pantothenate, 18 mg; nicotinic acid, 75 mg

### *Clostridium perfringens* challenge and sampling

*C. perfringens* challenge was conducted as originally developed by Dahiya et al. [19]. The particular organism, CVCC2027, was a type A field strain, isolated from a clinical case of NE in chickens, which did not carry the NetB gene, as determined by polymerase chain reaction (PCR). Briefly, the organism was cultured anaerobically on tryptose-sulphite-cycloserine agar base at 37 °C for 18 h, and then aseptically inoculated into cooked meat medium and incubated anaerobically at 37 °C overnight. All birds in challenged groups were orally gavaged in the crop once per day with 1.0 mL of actively growing *C. perfringens* culture from day 14 to 20 (1.0 × 10^8^ cfu/mL). On day 21 and 28, one bird per replicate was randomly selected and killed by intracardial administration of sodium pentobarbital (30 mg/kg body weight) and jugular exsanguination prior to sample collection.

### Intestinal lesion score

The small intestine from each bird was opened and scored blindly on a scale from zero to four as described by Dahiya et al. [19]: 0 = normal intestinal appearance with no lesion, 0.5 = severely congested serosa and mesentery engorged with blood, 1 = thin walled and friable intestines with small red petechiae (>5), 2 = focal necrotic lesions, 3 = patches of necrosis (1 to 2 cm-long), and 4 = diffused necrosis typical of field cases.

### Bacteriological examination

On day 21 and day 28, digesta for bacteriological examination were collected aseptically from ileum (from ileum midpoint to 2 cm proximal to ileocecal junction) and caecum, and stored at −80 °C. Bacterial populations were detected by the method of absolute quantitative real-time PCR (RT-PCR), as described by Wise and Siragusa, with some modifications [20]. Genomic DNA was isolated from 200 mg of digesta from ileum and caecum using a commercial kit (QIAamp DNA Stool Mini Kit, Qiagen Inc., Valencia, CA, USA). Extracted DNA was stored at −20 °C until analysis.

Standard curves for RT-PCR were prepared using DNA extracted from pure cultures to produce a high concentration of the target DNA by normal PCR amplification. Primer sequences were used in previous studies, which were designed on the basis of 16s rDNA sequences [21–23]. Target groups, primer sequences, amplicon sizes, and references are shown in Table 2. The targeted *Escherichia* subgroup contained genera of *E. coli*, *Hafnia alvei* and *Shigella* [22]. *E. coli* competent cells DH5α (Takara Bio Inc., Japan) were used to create plasmid standards. Firstly, PCR products were purified using a PCR purification kit (Biomed Gene Technologies, Beijing, China), and then cloned into pCR®2.1 using a TA cloning kit (Invitrogen Corporation, Carlsbad, CA, USA), following the manufacturer’s protocol. Purified insert-containing plasmids were quantified using a Nanodrop ND-1000 spectrophotometer (Thermo Fisher Scientific Inc., Waltham, MA, USA), and the number of target gene copies was calculated by the following formula according to Lee et al. [24]:

**Table 2:** 16s rDNA real-time PCR primers used to quantify intestinal bacteria

Target	Primer sequence (5′–3′) ^a^	Amplicon size (bp)	Reference
Total bacteria	F:ACTCCTACGGGAGGCAGCAGT	200	[19]
	R:GTATTACCGCGGCTGCTGGCAC		
*Lactobacillus* subgroup	F:AGCAGTAGGGAATCTTCCA	341	[19]
	R:CACCGCTACACATGGAG		
*Escherichia* subgroup ^b^	F:GTTAATACCTTTGCTCATTGA	340	[20]
	R:ACCAGGGTATCTAATCCTGT		
*Clostridium perfringens*	F:AAAGATGGCATCATCATTCAAC	279	[21]
	R:TACCGTCATTATCTTCCCCAAA		

^a^ F means forward, R means reverse

^b^ The targeted *Escherichia* subgroup contained genera of *E. coli*, *Hafnia alvei* and *Shigella*

1 $$ \mathrm{D}\mathrm{N}\mathrm{A}\left(\mathrm{copy}\right)=\frac{6.02\times {10}^{23}\left(\mathrm{copy}/\mathrm{mol}\right) \times \mathrm{D}\mathrm{N}\mathrm{A}\ \mathrm{amount}\left(\mathrm{g}\right)}{\mathrm{DNA}\ \mathrm{length}\ \left(\mathrm{d}\mathrm{p}\right) \times 660\ \left(\mathrm{g}/\mathrm{mol}/\mathrm{d}\mathrm{p}\right)} $$

Ten-fold serial dilutions of plasmid DNA were included on each 96-well plate to produce a standard curve. Genomic DNA from ileal samples and cecal samples was used as templates for absolute quantitative RT-PCR with a 7500 fluorescence detection system (Applied Biosystems, Foster City, CA, USA) according to optimized PCR protocols (SYBR-Premix Ex Taq, Takara Bio Inc., Japan). RT-PCR primers were the same as for normal PCR amplification primers (Table 2). Normal distributions were achieved by showing the results in terms of log_10_ gene copies/g intestinal digesta.

### Chemical analysis for thymol and carvacrol

On day 21, fresh digesta collected from small intestine (5 cm proximal to Meckel’s diverticulum) were acidified to pH < 2 with 2 % of 6 mol H_2_SO_4_ per litre to stop fermentation and stored at −20 °C pending analysis for thymol and carvacrol. Quantification of thymol and carvacrol in intestinal digesta was performed, as described by Michiels et al. with some modifications [25]. Briefly, 2.0 g of digesta from each bird were used for extraction, and 4 mL ethyl ethanoate was added as extraction solvent. All extractions were performed in duplicate. 2.5 mg of 2-isopropylphenol/mL of ethyl ethanoate was used as the internal standard. Ethyl ethanoate extracts were combined and reduced to dryness with nitrogen at room temperature. Then, the residue redissolved in ethyl ethanoate was used for gas chromatographic analysis.

### Statistical analysis

Results are given as mean values and pooled standard errors. Data were analyzed using the general linear model procedure of SAS software (SAS Institute Inc., Cary, NC, USA). The main effects of *C. perfringens* challenge, EO supplementation, and their interactive effects were analyzed. Duncan’s multiple-range tests were used to separate means when interactions were significant. When interactions were not significant, polynomial contrasts were conducted to determine linear and quadratic responses of the main-effect means (averaged between challenged and unchallenged groups) to dietary EO dosages. Statistical significance was considered at *P* ≤ 0.05, and 0.05 < *P* < 0.10 was considered a trend towards significance.

## Results

### In vitro antibacterial activity of EO and components

The antibacterial activities of EO and components (thymol and carvacrol) against selected pathogenic bacteria and beneficial *Lactobacillus* strains were expressed as MICs and MBCs (Table 3). Among the eight pathogenic bacteria, *E. coli* was more sensitive to thymol (MIC and MBC, 187.5 and 375 μg/mL, respectively). In contrast, *S.* Enteritidis was more sensitive to carvacrol (MIC and MBC, 187.5 and 750 μg/mL, respectively). However, the greatest MIC and MBC were obtained for thymol in the presence of *S.* Enteritidis (750 and 1500 μg/mL, respectively). Thymol and carvacrol exhibited the same antibacterial activity against *C. perfringens, S.* Typhimurium and *S.* Pullorum (MIC and MBC, 375 and 750 μg/mL, respectively). Moreover, the tested EO product showed identical antibacterial activity against all eight pathogenic bacteria (MIC and MBC, 750 and 1500 μg/mL, respectively). In addition, antibacterial activity of thymol and carvacrol was noticed against *L. acidophilus*, *L. reuteri* and *L. salivarius* at concentrations higher than for pathogenic bacteria (MIC and MBC, 1500 and 3000 μg/mL, respectively). The EO was also observed to inhibit *Lactobacillus* growth (MIC and MBC, both 3000 μg/mL). In addition, the FICI towards all tested bacteria fell between 0.5 and 1.0, demonstrating an additive antibacterial effect between thymol and carvacrol.

**Table 3:** Antibacterial activity of essential oils and components towards selected bacteria

Bacteria	MIC (μg/mL)			MBC (μg/mL)			FICI
	EO	Thymol	Carvacrol	EO	Thymol	Carvacrol	
*Escherichia coli*	750	187.5	375	1500	375	750	0.5
*Clostridium perfringens*	750	375	375	1500	750	750	0.5
*Salmonella* Typhimurium	750	375	375	1500	750	750	0.5
*Salmonella* Enteritidis	750	750	187.5	1500	1500	750	1.0
*Salmonella* Pullorum	750	375	375	1500	750	750	0.5
*Lactobacillus acidophilus*	3000	1500	1500	3000	3000	3000	1.0
*Lactobacillus reuteri*	3000	1500	1500	3000	3000	3000	1.0
*Lactobacillus salivarius*	3000	1500	1500	3000	3000	3000	1.0

MIC, the minimum inhibitory concentration; MBC, the minimal bactericidal concentration; EO, essential oil contained 25 % thymol and 25 % carvacrol as active components; FICI, fractional inhibitory concentration index

### Intestinal lesion score

**Fig. 1:**
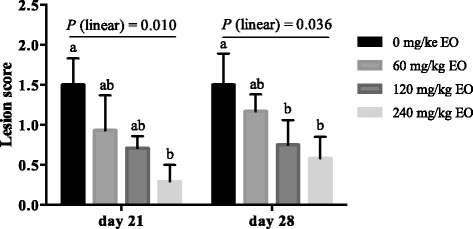
Effects of essential oils on intestinal lesion score of *Clostridium perfringens* challenged broiler chickens. Values are means of eight replicates per treatment and pooled standard error of the mean. Bars not sharing a common letter differ significantly (*P* ≤ 0.05). EO, essential oils; *P* (linear), polynomial contrasts were conducted to determine the linear response of lesion score to dietary EO dosages

No intestinal lesions were observed in unchallenged birds. In challenged groups, intestinal lesion scores were reduced linearly with increased dietary EO dosages on day 21 and day 28 (*P* = 0.010 and 0.036, respectively, Fig. 1). Compared with birds fed basal diet, lesion severity in broilers fed 240 mg/kg EO was reduced significantly on day 21 (*P* ≤ 0.05), and lesion severity of broilers fed 120 and 240 mg/kg EO was reduced significantly on day 28 (*P* ≤ 0.05).

### Quantitation of ileal and cecal microbiota

*C. perfringens* challenge led to significant increases in ileal populations of *Escherichia* subgroup (*P* < 0.001) and *C. perfringens* (*P* = 0.03) on day 1 post-challenge (Table 4). However, on day 7 post-challenge, ileal microbiota of challenged birds were restored to the level of unchallenged birds (*P* > 0.10, Table 5). Dietary supplementation of EO did not influence *C. perfringens* numbers, but linearly decreased *Escherichia* numbers in ileum on day 21 and 28 (*P* = 0.027 and 0.071, respectively) regardless of challenge (Tables 4 and 5). For cecal microbiota, the population of *C. perfringens* was increased on day 1 post-challenge (*P* < 0.001, Table 6), and showed a tendency to increase on day 7 post-challenge (*P* = 0.078, Table 7). Dietary EO supplementation influenced cecal *Lactobacillus* numbers quadratically (*P* = 0.002) on day 21 (Table 6). Compared with birds fed basal diet, higher *Lactobacillus* populations were observed in birds fed 60 mg/kg and 120 mg/kg EO on day 21 in both unchallenged and challenged groups (Table 6). In contrast, the populations of total bacteria and *Escherichia* in caecum decreased linearly with increased EO dosages on day 28 (*P* = 0.026 and 0.060, respectively, Table 7). No interactions were observed for ileal and cecal microbiota between *C. perfringens* challenge and EO supplementation (*P* > 0.10, Table 4, 5, 6 and 7).

**Table 4:** Effect of essential oils on ileal bacterial populations of broilers on day 21

Item ^a^	Total bacteria	*Lactobacillus*	*Escherichia*	*C. perfringens*
Treatment				
Basal diet, unchallenged	9.00	7.89	8.00	2.27
60 mg/kg EO, unchallenged	9.28	7.98	7.60	2.35
120 mg/kg EO, unchallenged	9.35	8.30	7.55	2.34
240 mg/kg EO, unchallenged	9.22	8.14	7.15	2.34
Basal diet, challenged	9.41	8.11	8.72	2.50
60 mg/kg EO, challenged	9.48	8.38	8.99	2.39
120 mg/kg EO, challenged	9.44	8.39	8.26	2.43
240 mg/kg EO, challenged	9.29	8.27	8.03	2.57
SEM	0.068	0.073	0.137	0.033
*P*-value				
Challenge	0.185	0.169	<0.001	0.030
EO	0.735	0.438	0.095	0.805
Challenge × EO	0.828	0.888	0.713	0.655
Linear ^b^	0.945	0.366	0.027	0.553
Quadratic ^b^	0.261	0.172	0.853	0.707

^a^ Values are means of eight replicates per treatment, and expressed as log_10_ (copy/g digesta). EO, essential oils; unchallenged, birds without challenge of *C. perfringens*; challenged, birds challenged with *C. perfringens* from day 14 to 20; SEM, pooled standard error of the mean; Challenge × EO, interaction between *C. perfringens* challenge and EO supplementation

^b^ When interaction between *C. perfringens* challenge and EO supplementation was not significant, linear and quadratic polynomial contrasts were performed on the main-effect means to EO dosages

**Table 5:** Effect of essential oils on ileal bacterial populations of broilers on day 28

Item ^a^	Total bacteria	*Lactobacillus*	*Escherichia*	*C. perfringens*
Treatment				
Basal diet, unchallenged	9.13	7.70	6.09	3.00
60 mg/kg EO, unchallenged	9.08	7.76	6.27	2.79
120 mg/kg EO, unchallenged	8.85	7.71	5.90	2.94
240 mg/kg EO, unchallenged	9.13	7.76	5.91	2.84
Basal diet, challenged	9.26	7.99	6.13	2.96
60 mg/kg EO, challenged	9.33	8.18	6.56	3.04
120 mg/kg EO, challenged	9.08	7.98	5.76	2.95
240 mg/kg EO, challenged	8.91	7.80	5.60	2.95
SEM	0.058	0.081	0.098	0.051
*P*-value				
Challenge	0.421	0.135	0.887	0.462
EO	0.365	0.877	0.092	0.953
Challenge × EO	0.469	0.882	0.742	0.789
Linear ^b^	0.215	0.662	0.071	0.619
Quadratic ^b^	0.502	0.708	0.780	0.931

^a^ Values are means of eight replicates per treatment, and expressed as log_10_ (copy/g digesta). EO, essential oils; unchallenged, birds without challenge of *C. perfringens*; challenged, birds challenged with *C. perfringens* from day 14 to 20; SEM, pooled standard error of the mean; Challenge × EO, interaction between *C. perfringens* challenge and EO supplementation

^b^ When interaction between *C. perfringens* challenge and EO supplementation was not significant, linear and quadratic polynomial contrasts were performed on the main-effect means to EO dosages

**Table 6:** Effect of essential oils on cecal bacterial populations of broilers on day 21

Item ^a^	Total bacteria	*Lactobacillus*	*Escherichia*	*C. perfringens*
Treatment				
Basal diet, unchallenged	11.16	8.52	9.77	3.05
60 mg/kg EO, unchallenged	11.50	8.86	9.53	2.38
120 mg/kg EO, unchallenged	11.41	9.16	9.51	2.83
240 mg/kg EO, unchallenged	11.42	8.69	9.41	2.41
Basal diet, challenged	11.28	8.60	9.28	3.68
60 mg/kg EO, challenged	11.43	9.02	9.42	3.63
120 mg/kg EO, challenged	11.40	9.06	9.46	3.30
240 mg/kg EO, challenged	11.37	8.70	9.34	3.39
SEM	0.041	0.068	0.085	0.110
*P-value*				
Challenge	0.973	0.785	0.316	<0.001
EO	0.190	0.022	0.952	0.327
Challenge × EO	0.842	0.916	0.783	0.466
Linear ^b^	0.299	0.673	0.566	0.270
Quadratic ^b^	0.129	0.002	0.927	0.606

^a^ Values are means of eight replicates per treatment, and expressed as log_10_ (copy/g digesta). EO, essential oils; unchallenged, birds without challenge of *C. perfringens*; challenged, birds challenged with *C. perfringens* from day 14 to 20; SEM, pooled standard error of the mean; Challenge × EO, interaction between *C. perfringens* challenge and EO supplementation

^b^ When interaction between *C. perfringens* challenge and EO supplementation was not significant, linear and quadratic polynomial contrasts were performed on the main-effect means to EO dosages

**Table 7:** Effect of essential oils on cecal bacterial populations of broilers on day 28

Item ^a^	Total bacteria	*Lactobacillus*	*Escherichia*	*C. perfringens*
Treatment				
Basal diet, unchallenged	11.18	9.14	8.83	2.68
60 mg/kg EO, unchallenged	11.19	9.10	8.66	2.54
120 mg/kg EO, unchallenged	11.31	9.16	8.80	2.55
240 mg/kg EO, unchallenged	11.00	8.99	8.55	2.42
Basal diet, challenged	11.18	9.22	9.24	2.79
60 mg/kg EO, challenged	11.24	9.30	9.01	3.24
120 mg/kg EO, challenged	10.89	9.19	8.77	2.78
240 mg/kg EO, challenged	10.81	9.02	8.42	2.47
SEM	0.046	0.056	0.092	0.077
*P*-value				
Challenge	0.126	0.473	0.428	0.078
EO	0.076	0.624	0.269	0.205
Challenge × EO	0.220	0.951	0.685	0.406
Linear ^b^	0.026	0.255	0.060	0.112
Quadratic ^b^	0.399	0.509	0.928	0.437

^a^ Values are means of eight replicates per treatment, and expressed as log_10_ (copy/g digesta). EO, essential oils; unchallenged, birds without challenge of *C. perfringens*; challenged, birds challenged with *C. perfringens* from day 14 to 20; SEM, pooled standard error of the mean; Challenge × EO, interaction between *C. perfringens* challenge and EO supplementation

^b^ When interaction between *C. perfringens* challenge and EO supplementation was not significant, linear and quadratic polynomial contrasts were performed on the main-effect means to EO dosages

### Thymol and carvacrol concentrations in intestinal digesta

Thymol and carvacrol concentrations in intestinal digesta linearly increased with increased dietary EO dosages (*P* < 0.001, Table 8). For birds fed 240 mg/kg EO, thymol and carvacrol accounted for 60 mg/kg, as each comprised 25 % of the EO product. Their average concentrations in intestinal digesta were 0.80 and 0.71 μg/g (based on digesta wet weight) respectively, which were significantly higher than concentrations assayed in birds fed 60 and 120 mg/kg EO (*P ≤* 0.05).

**Table 8:** Analytical concentrations of thymol and carvacrol in intestinal digesta

Item ^a^	Dietary EO concentration (mg/kg)				SEM	*P*-value	Linear ^b^	Quadratic ^b^
	0	60	120	240				
Thymol (μg/g digesta)	ND	0.21^B^	0.46^B^	0.80^A^	0.076	<0.001	<0.001	0.586
Carvacrol (μg/g digesta)	ND	0.20^B^	0.29^B^	0.71^A^	0.076	<0.001	<0.001	0.677

^a^ Values are means of eight replicates per treatment. Means within the same row not sharing a common uppercase superscript letter differ significantly (*P* ≤ 0.05). EO, essential oil contained 25 % thymol and 25 % carvacrol as active components; SEM, pooled standard error of the mean; ND, not detected

^b^ Linear and quadratic responses of intestinal thymol or carvacrol concentrations to dietary EO dosages

## Discussion

EO and their purified constituents are promising alternatives to growth-promoting antibiotics, and they are generally recognized as safe [6]. Among thousands of EO constituents, thymol and its isomer carvacrol are the main components of commonly used oregano and thyme oil [26]. In the present study, strong antibacterial effects of an EO product and its components thymol and carvacrol were observed in vitro against a panel of pathogenic bacteria, including *E. coli*, *Salmonella,* and *C. perfringens*. In broiler chickens challenged with *C. perfringens*, dietary supplementation of thymol and carvacrol-bearing EO did not influence *C. perfringens* numbers. However, EO did alter *Escherichia* and *Lactobacillu* populations, and alleviated intestinal lesions in a linear dose-related manner. In addition, thymol and carvacrol concentrations in intestinal digesta were assayed in this study.

Many studies have tested the antibacterial activity of EO, or their constituents, against foodborne pathogens [11, 16, 26–30]. According to Pei et al., MICs of thymol and carvacrol against *E. coli* were both 400 μg/mL [26]. And Ivanovic et al. have reported that *E. coli* and *S.* Enteritidis are inhibited by thymol at concentrations between 160 and 320 μg/mL [16]. Also, *C. perfringens* was inhibited effectively by thymol and carvacrol (MICs of 240 and 300 μg/mL, respectively) [29, 31]. In the present study, strong antibacterial effects of thymol and carvacrol were observed against selected pathogenic bacteria, and similar MICs of thymol and carvacrol were obtained to previous reports. In addition, the *E. coli* strains we used were more sensitive to thymol, while the *S.* Enteritidis strains we used were more sensitive to carvacrol, compared with other pathogens. Besides, the antibacterial activity of the EO product was comparable to thymol and carvacrol according to the present work, taking into account that the EO product contained 25 % each of thymol and carvacrol as active components.

However, limited information is available regarding EO effects on beneficial probiotic bacteria, such as *Lactobacillus*. Only slight or no inhibition against *Lactobacillus* has been reported for carvacrol at 300 μg/mL, according to Si et al. [31]. In the present study, higher concentrations of thymol and carvacrol (up to 6000 μg/mL) were used to explore their bactericidal activities. The growth of *L. acidophilus*, *L. reuteri* and *L. salivarius* was observed to be inhibited by thymol and carvacrol at an MIC of 1500 μg/mL, and be killed at twice the concentration, the MBC. Also, the EO product was able to kill the tested *Lactobacillus* strains at 3000 μg/mL. Ouwehand et al. have pointed out that *L. fermentum* and *L. reuteri* growth is inhibited by thymol itself as well as by EO (oregano, rosemary and thyme oils, whose main constitutes were thymol and/or carvacrol) at 500 μg/mL [32]. However, no antibacterial effects against *L. plantarum* were found for carvacrol even at 3000 μg/mL in Ben Arfa et al.’s study [9]. The contrary results observed here might be explained by differences in the examined *Lactobacillus* field strains. In addition, the final concentrations of microbial suspensions in the present study were 1-5 × 10^5^ cfu/mL for the MIC assays, but a different range of 10^6^–10^7^ cfu/mL was employed in Ben Arfa et al.’s study [9]. Nevertheless, the present results showed that *Lactobacillus* appeared more resistant to EO and its constitutes than pathogenic bacteria did, which might indicate that intestinal microbiota in animals might be favored under certain concentrations of EO and its constituents.

According to in vitro experiments, additive antibacterial effects between thymol and carvacrol were observed towards all tested bacteria, as the FICI was between 0.5 and 1.0, and FICI was applied to define the nature of interaction. However, FICI values used for the definition of interaction differed between publications, which makes comparison of studies difficult [33]. The definition we adopted in the present study appears to be more acceptable in literature [33]. Based on the definition in the present study, an additive effect (FICI of 0.75) between thymol and carvacrol against *E. coli* also has been observed in previous studies [26, 27]. Similarly, Lambert et al. have reported that thymol and carvacrol in combination show additive effects against *Pseudomonas aeruginosa* and *Staphylococcus aureus* using half-fold dilutions within a Bioscreen plate [34]. In addition, the additive effects observed here were in agreement with the antibacterial activity of the EO product, whose MIC and MBC were comparable to its active components thymol and carvacrol. This would be expected as thymol and carvacrol are isomers with similar chemical structures and likely to have similar mechanisms of antimicrobial activity [35].

In in vivo trial, wheat-based diets were used to favor *C. perfringens* colonization. However, the challenge did not result in overtly clinical signs of NE or NE-related mortality in the present trial. The deficiency of NetB gene in the *C. perfringens* strain used here might partially explain the loss of characteristic NE, as NetB has been observed to play a major role in NE [3, 36]. Despite this, *C. perfringens* challenge damaged intestinal mucosa, as observed by macroscopic lesions, which was similar to previous studies using the same challenge model to create sub-clinical NE [37, 38]. And dietary EO supplementation alleviated intestinal lesions linearly on day 21 and 28. This was in agreement with previous studies, which have reported that thymol and carvacrol-bearing EO alleviates *C. perfringens-*induced gut lesions [29, 39].

The abundance of total bacteria, *Lactobacillus*, *Escherichia,* and *C. perfringens* in the gut was estimated using absolute RT-PCR to evaluate group-specific 16s rDNA in extracted community DNA of intestinal digesta. According to Dahiya et al., *C. perfringens* numbers can reach 10^7^–10^9^ cfu/g digesta in typical clinical NE [5], but *C. perfringens* numbers were lower than 10^4^ cfu/g digesta in the present study. Even though, microbial populations dramatically changed when birds were infected with *C. perfringens*, with *C. perfringens* populations significantly increased in both ileum and caecum on day 1 post-challenge. These changes were also accompanied by a significant *Escherichia* increase in ileum. Many populations of bacteria have been reported to coexist with NE and one of the largest populations is *E. coli* [40]. This was consistent with the results obtained in the current study as well as other work performed by Liu et al. [38].

The results of the present work indicated that dietary supplementation of EO linearly decreased ileal *Escherichia* counts on day 21 and 28, and linearly decreased the number of total bacteria in caecum on day 28. In addition, *Lactobacillus* counts were quadratically affected by dietary EO addition in caecum on day 21. Similarly, Jamroz et al. have reported that the combination of carvacrol, cinnamaldehyde and capsaicin decreases *E. coli* populations and increase *Lactobacillus* numbers in broilers fed a wheat-based diet [14]. Also, EO extracted from thyme and anise have been reported to decrease *C. perfringens* and *E. coli* counts in both small and large intestines, accompanied by decreased intestinal lesion score [41]. Changes in intestinal microbiota might be related to alleviation of intestinal lesions with EO supplementation. Interestingly, no inhibition of *C. perfringens* was observed for thymol and carvacrol in the present study. This was inconsistent with results obtained by Jamroz et al. and Cho et al. regarding the effects of EO on *C. perfringens* [14, 41]. Notably, in Abildgaard et al.’s study, *C. perfringens* numbers were not influenced by dietary supplementation of an EO product (containing thymol, eugenol, curcumin, and piperin) [42]. In fact, different from the well-known antibacterial activities of EO in vitro, their in vivo effects on intestinal bacteria are rather limited with variable results. For example, no alteration in intestinal microbiota was observed in broilers fed an EO-supplemented diet according to Cross et al. and Hong et al. [12, 13]. As mentioned above, EO blends containing various constituents have been used in vivo in published reports, making it difficult to compare their efficacy [6, 8, 12–15].

The antibacterial efficacy of EO is related to the concentrations of active components. However, studies have shown that most EO are absorbed quickly after oral administration. According to Kohlert et al., peak plasma thymol concentration was reached after 2 h in humans [43]. In piglets, plasma thymol and carvacrol concentrations peaked at 1.39 and 1.35 h, respectively, and thymol and carvacrol were almost completely absorbed in stomach and proximal small intestine [25]. To exert antibacterial activity in vivo, it has been suggested that delivery protection is needed to aid EO in reaching target sites within the gut [44, 45]. In the present study, the EO product was stabilized with glycerides. Since little information is available regarding the release of active EO components in broiler intestine, thymol and carvacrol concentrations in intestinal digesta were measured in the present study. It was observed that intestinal thymol and carvacrol concentrations increased linearly with increased dietary EO dosages. The highest concentrations observed for thymol and carvacrol were 0.80 and 0.71 μg/g, respectively, in broilers fed 240 mg/kg EO. This was in accordance with the linear inhibition effects of EO on *Escherichia* in broilers. Similarly, Michiels et al. have investigated thymol and carvacrol concentrations in piglet gastrointestinal digesta [46]. They observed 5 μg/g carvacrol in the proximal small intestine of piglets fed 2 g/kg carvacrol diet, and 13 ~ 24 μg/g thymol in piglets fed 2 g/kg thymol diet [46]. Although the EO used here was coated with glycerides, the analyzed luminal concentrations of thymol and carvacrol were quite low (much lower than their MICs in vitro), which may partially explain the unchanged *C. perfringens* populations in vivo. To our knowledge, this is the first time that in vitro antibacterial activities and luminal availability of active EO constituents in chicken were investigated, providing a link between EO activities in vitro and their effects in vivo. According to our finding, more efficient protection and release techniques are necessary to improve luminal availability of EO constituents.

Interestingly, some reports have shown that sub-lethal EO concentrations have important effects on bacterial activity [47, 48]. According to Inamuco et al., at concentrations where growth of *Salmonella* was not inhibited, carvacrol completely inhibited *Salmonella* motility, and reduced its invasion of porcine intestinal epithelial cells (47). In another study, carvacrol did not influence the motility of *Bacillus cereus*, but significantly inhibited toxin production at doses below its MIC [48]. In the present study, intestinal lesions were alleviated linearly with increased EO dosages despite of unaffected *C. perfringens* populations. These results suggest that thymol and carvacrol might influence *C. perfringens* adhesion to intestinal mucosa or decrease toxin production without affecting its numbers, which need further studies to be verified. Furthermore, previous studies have shown that supplemental EO can enhance cellular and humeral immunity in chickens, which might be involved in affecting intestinal microbiota and gut health [49, 50].

## Conclusions

The present study showed that thymol and carvacrol possessed strong antibacterial activity against pathogenic bacterial strains and weak activity towards beneficial *Lactobacillus* strains in vitro. In addition, an additive effect was found when thymol and carvacrol were applied in combination. In broiler chickens challenged with *C. perfringens*, dietary supplementation of thymol and carvacrol-bearing EO alleviated intestinal lesions, which might be related to changes in intestinal microbiota. More specific studies are required to improve luminal availability of EO constituents, and clarify how EO affect intestinal microbiota in vivo*.*

## Abbreviations

cfu: Colony-forming units
DMSO: Dimethylsulfoxide
EO: Essential oils
FIC: Fractional inhibitory concentration
FICI: Fractional inhibitory concentration index
IU: International unit
MBC: Minimal bactericidal concentration
MIC: Minimum inhibitory concentration
MRS: Man-Rogosa-Sharpe
NE: Necrotic enteritis
OD: Optical density
PCR: Polymerase chain reaction
RT-PCR: Quantitative real-time PCR
